# Culture Dimensionality Shapes Drug Sensitivity to Kinase Inhibitor Combinations with Epigenetic or Metabolic Inhibition in a Hepatocellular Carcinoma Model

**DOI:** 10.3390/biomedicines14102326

**Published:** 2026-10-13

**Authors:** Claudia Kunst, Tobias Schilling, Cedric Arling, Manuela Peisl-Gunckel, Sophie Schlosser-Hupf, Karsten Gülow, Martina Müller

**Affiliations:** 1 Department of Internal Medicine I, Gastroenterology, Hepatology, Endocrinology, Rheumatology, Immunology and Infectious Diseases, University Hospital Regensburg, 93053 Regensburg, Germany; cedric.arling@gmail.com (C.A.); manuela.gunckel@ukr.de (M.P.-G.); sophie.schlosser-hupf@ukr.de (S.S.-H.); karsten.guelow@ukr.de (K.G.); martina.mueller-schilling@ukr.de (M.M.); 2 Department of Emergency and Intensive Care Medicine, Klinikum Stuttgart, 70174 Stuttgart, Germany; t.schilling@klinikum-stuttgart.de

**Keywords:** hepatocellular carcinoma, 3D culture, combination treatment, tyrosine kinase inhibition, sorafenib, regorafenib, HDAC (histone deacetylase) inhibition, panobinostat, PHGDH (phosphoglycerate dehydrogenase) inhibition, NCT-503

## Abstract

**Background/Objectives:** Hepatocellular carcinoma (HCC) remains one of the deadliest malignancies worldwide, and treatment efficacy is often limited by tumor heterogeneity and therapy resistance. Preclinical models, particularly three-dimensional (3D) cell cultures, are increasingly used to better mimic the tumor microenvironment and improve the predictive value of drug testing. This study aimed to compare the effectiveness of different pharmacological treatments for HCC in two-dimensional (2D) monolayer and 3D spheroid HepG2 culture models and to investigate the underlying cell death mechanisms. The multikinase inhibitors sorafenib and regorafenib, the epigenetic histone deacetylase (HDAC) inhibitor panobinostat, and the metabolic phosphoglycerate dehydrogenase (PHGDH) inhibitor NCT-503 were evaluated as single agents and in combination. **Results:** All tested compounds induced cell death and reduced cellular ATP levels in both 2D and 3D systems. Combination treatments of sorafenib or regorafenib with either panobinostat or NCT-503 generally showed no significant deviation from additivity in cytotoxicity and cellular energy levels, although significant sub-additive deviations were observed for some panobinostat combinations in the ATP analyses. Mechanistically, panobinostat induced apoptotic cell death, as indicated by caspase-3 and PARP cleavage, increased caspase-3/7 activity, and inhibition by the pan-caspase inhibitor zVAD-FMK, whereas NCT-503-mediated cell death occurred largely independently of canonical caspase-mediated apoptosis in 2D cultures. Although similar effects were observed in 3D spheroids, cell death responses were attenuated compared with 2D cultures, whereas reductions in cellular ATP levels remained comparable, indicating a differentiated relationship between ATP depletion and cell death in 2D and 3D cultures. **Conclusions:** In summary, combining tyrosine kinase inhibition with epigenetic or metabolic targeting agents increased cell death and impaired cellular energy homeostasis more effectively than single-agent treatment in HepG2 cells, with no significant deviation from additivity detected in most combination analyses. Notably, 3D-cultured HepG2 cells showed attenuated cell death responses despite comparable reductions in cellular ATP levels, highlighting differences in drug responses between 2D and 3D culture models. These findings support the use of 3D culture models as a complementary approach to conventional 2D systems for evaluating drug responses, particularly as they reveal an attenuated cell death response compared with 2D cultures.

## 1. Introduction

Hepatocellular carcinoma (HCC) is the most common primary malignancy of the liver and a major global health burden. In 2020, malignant liver tumors were the sixth most frequently diagnosed and the third leading cause of cancer-related death worldwide, with approximately 906,000 new cases and 830,000 deaths. HCC affects men 2–3 times more frequently than women in most regions, including Europe, with particularly high incidence rates in East and Southeast Asia as well as in North and West Africa. Globally, HCC accounts for 75–95% of all primary liver cancers [1,2,3,4,5].

HCC arises from a complex interplay of environmental, genetic, and epigenetic factors that drive tumor progression and therapy resistance [5,6]. While chronic infections with hepatitis B and C viruses have historically been the primary drivers of HCC, the disease etiology is shifting, with metabolic syndrome-related conditions (e.g., diabetes), alcohol-related liver disease, and metabolic dysfunction-associated steatotic liver disease (MASLD) emerging as leading risk factors in Western countries and gaining recognition globally [2,7,8]. Less common hereditary disorders, such as hemochromatosis or alpha-1-antitrypsin deficiency, and exposure to aflatoxins can also increase risk [2]. Irrespective of the underlying cause, HCC continues to have a poor prognosis, with an overall 5-year survival rate of around 18% [9,10,11,12].

A major challenge in HCC is that early-stage disease is often asymptomatic, resulting in late diagnosis when curative-intent options are no longer feasible. Treatment decisions are guided by the Barcelona Clinic Liver Cancer (BCLC) staging system, which incorporates tumor burden, liver function, and cancer-related symptoms [2,6,13,14,15]. For patients with early-stage disease, curative therapeutic options, including resection, ablation, or transplantation, are available, with tumor recurrence remaining frequent. Intermediate and advanced stages are managed with locoregional or systemic therapies [14,16].

Building on these clinical challenges, current first-line systemic therapies for HCC are guided by EASL recommendations and include immune checkpoint inhibitor-based approaches and tyrosine kinase inhibitors [2,14]. Recommended combinations therapies comprise atezolizumab plus bevacizumab, tremelimumab plus durvalumab, camrelizumab plus rivoceranib, ipilimumab plus nivolumab, and sintilimab plus bevacizumab biosimilar (IBI305) [14,17,18,19,20,21,22,23].

For patients ineligible for immunotherapy, tyrosine kinase inhibitors such as lenvatinib and sorafenib remain first-line treatment options. They were, until recently, the only agents available for HCC [2,14,24,25,26,27,28,29]. Sorafenib, the first approved multikinase inhibitor for this indication, improved median survival from 7.9 to 10.7 months compared with the placebo in the SHARP trial [29]. However, intrinsic and acquired resistance limit its long-term efficacy [30,31,32]. Lenvatinib, as a multikinase inhibitor, subsequently demonstrated non-inferior overall survival compared with sorafenib [28].

Tyrosine kinase inhibitors, such as lenvatinib, sorafenib, regorafenib, and cabozantinib continue to play an essential role in second-line therapy, while ramucirumab remains an important option for patients with AFP ≥ 400 ng/mL. Similar to sorafenib, regorafenib is an oral multikinase inhibitor targeting several oncogenic and angiogenic pathways [2,33].

HCC remains a major clinical challenge because of its marked molecular heterogeneity and the frequent development of resistance to conventional therapies. Resistance to apoptosis represents a fundamental hallmark of cancer biology and is likewise a characteristic feature of HCC [34]. Overcoming intrinsic and acquired resistance may thus depend on the coordinated targeting of central survival networks in order to activate multiple cell death pathways.

Histone deacetylase (HDAC) inhibitors are epigenetic modulators that alter chromatin structure and gene expression, thereby affecting proliferation, cell cycle control, and cell death pathways [35]. By engaging apoptotic signaling and increasing tumor cell sensitivity to anticancer agents, HDAC inhibition has shown synergistic potential when combined with targeted therapies [36,37]. Panobinostat (LBH589), a potent pan-HDAC inhibitor, exerts antitumor effects in HCC models by inducing apoptosis and autophagy and suppressing angiogenic signaling [36,37,38]. These properties provide a strong rationale for combining HDAC inhibitors with multikinase inhibitors to simultaneously target proliferative signaling and survival mechanisms in HCC.

NCT-503 is a selective metabolic inhibitor of phosphoglycerate dehydrogenase (PHGDH), the rate-limiting enzyme of de novo serine biosynthesis. Serine metabolism supports redox homeostasis and contributes to therapy resistance [39,40]. In HCC, sorafenib induces PHGDH, mitigating oxidative stress from reactive oxygen species (ROS) and reducing drug sensitivity [41]. Preclinical studies show that NCT-503 enhances anticancer efficacy across tumor types. It enhances the effects of sorafenib in HCC [41], reverses PHGDH-driven ferroptosis resistance in prostate cancer [42], and induces ROS-dependent apoptosis in non-small cell lung cancer (NSCLC) via p53/p21 and caspase-3/PARP activation [43]. In glioblastoma, PHGDH inhibition sensitizes cells to radiotherapy [44]. Similar effects have been reported in triple-negative breast cancer and colon cancer organoids [45,46].

Traditional two-dimensional (2D) cell cultures often fail to recapitulate the complex architecture, cell–cell interactions, and microenvironment of tumors, limiting their predictive value for drug testing. Three-dimensional (3D) culture systems, including spheroids and organoids, better mimic in vivo tissue organization, gradients of nutrients and oxygen, and cellular heterogeneity. These models have been shown to influence drug penetration, efficacy, and resistance mechanisms, providing a more physiologically relevant platform for evaluating anticancer agents and elucidating therapeutic responses [47,48].

The aim of this study was to assess whether 3D spheroid models provide a more physiologically relevant platform than conventional 2D cultures for evaluating drug response in HCC. We compared the effects of individual drugs and combination treatments in both 2D monolayer cultures and 3D spheroid models. By examining treatment responses across these systems, we sought to determine how culture dimensionality influences drug sensitivity and resistance mechanisms, and to inform the selection of appropriate in vitro models for HCC drug testing.

## 2. Materials and Methods

### 2.1. Cell Culture

The human HepG2 cell line (American Type Culture Collection (ATCC) HB-8065, Manassas, VA, USA) was cultured in RPMI-1640 medium (Anprotec, Bruckberg, Germany) supplemented with 10% fetal bovine serum (PanReac AppliChem, Darmstadt, Germany) without antibiotics at 37 °C in a humidified atmosphere of 5% CO_2_.

For three-dimensional cell culture in spheroids, cells were harvested from two-dimensional cultures, resuspended in culture medium, and counted. Subsequently, 7000 cells were seeded into each well of a round-bottom 96-well plate and centrifuged at 250× *g* for 2 min. The plates were then incubated at 37 °C for 24 h.

### 2.2. Cell Treatment with Compounds

HepG2 cells were exposed for 48 h to sorafenib (TargetMol, Wellesley Hills, MA, USA) at 5–10 µM, regorafenib (Selleck Chemicals, Houston, TX, USA) at 5–10 µM, panobinostat (Selleck Chemicals, Houston, TX, USA) at 0.02–0.5 µM, and NCT-503 (Hölzel Diagnostika Handels GmbH, Cologne, Germany) at 40–80 µM. With 5 and 10 µM, concentrations of sorafenib and regorafenib were selected to encompass the range around or above the reported IC_50_ values in HepG2 cells [49,50]. Panobinostat was tested at 0.02 µM, close to the clinically reported plasma concentrations, and at 0.5 µM to assess responses at higher pharmacological exposure [51]. A total of 40 µM NCT-505 was selected based on previous studies in HCC models, which reported a GI_50_ of approximately 50 µM and used 40 µM in combination with sorafenib [41]. A higher concentration of 80 µM was included to assess dose-dependent effects. Compounds were administered either individually or in combination. All compounds were dissolved in DMSO. DMSO was used as the vehicle control, and its final concentration matched across treatment and control conditions. Experiments were repeated 3–5 times as independent biological replicates. Technical replicates within each experiment were averaged before statistical analysis and were not treated as independent observations.

### 2.3. Cell Death Analysis

Cell death was quantified by Annexin V/DAPI exclusion staining. Cells were harvested 48 h after treatment with the respective compound(s), stained with Annexin V–FITC and DAPI (BD Biosciences, Heidelberg, Germany) according to the manufacturer’s instructions, and analyzed by flow cytometry using an LSRFortessa™ Cell Analyzer (BD Biosciences, San Jose, CA, USA). Viable cells were defined as Annexin V^−^/DAPI^−^.

To ensure comparability across independent experiments, specific cell death was calculated using the following formula [52]:

$$\mathrm{Specific cell death}\left(\%\right)=\frac{\mathrm{experimental cell death}\left(\%\right)-\mathrm{spontaneous cell death}\left(\%\right)}{100-\mathrm{spontaneous cell death}\left(\%\right)}\times 100$$

### 2.4. ATP-Based Cell Viability Assay

Cell viability was measured via ATP using CellTiter-Glo assays (Promega, Madison, WI, USA). For 2D cultures, 7000 cells per well were seeded in 96-well plates and incubated for 24 h, followed by compound treatment in triplicate for 48 h. After adding 100 µL of reagent per well and 10 min incubation with shaking, luminescence was recorded. For 3D cultures, viability was assessed using the CellTiter-Glo^®^ 3D Assay following the same procedure.

### 2.5. Caspase Inhibition by zVAD-FMK

For the pharmacological inhibition of caspase activity, the cell-permeable irreversible pan-caspase inhibitor zVAD-FMK (Z-Val-Ala-Asp(OMe)-fluoromethyl ketone; Selleck Chemicals, Houston, TX, USA) was used. A 50 mM stock solution was prepared in DMSO, aliquoted, and stored at −20 °C. Cells were pretreated with zVAD-FMK (50 µM) for 20 min prior to incubation with the respective compounds and maintained in the presence of the inhibitor throughout the experiment (48 h). An equivalent final concentration of DMSO was used for the vehicle controls.

### 2.6. Western Blot

For protein isolation, cells were washed with PBS and lysed in RIPA buffer containing protease and phosphatase inhibitors (cOmplete ULTRA; Sigma Aldrich, St. Louis, MO, USA). Lysates were incubated on ice for 20 min and centrifuged at 16,000 rpm for 45 min at 4 °C. Protein-containing supernatants were collected, separated by SDS–PAGE, and transferred to PVDF membranes.

Antibodies detecting full-length and cleaved caspase-3 (#9662, Cell Signaling Technology, Danvers, MA, USA) were used at 1:1000 dilution. β-actin (Sigma-Aldrich, St. Louis, MO, USA) was used as a loading control. Secondary antibodies against rabbit or mouse IgG (Sigma-Aldrich, St. Louis, MO, USA) were used at 1:10,000 dilution.

### 2.7. Caspase-3/7 Activity Assay

Caspase-3/7 activity was measured using the Caspase-Glo^®^ 3/7 Assay (Promega Corporation, Madison, WI, USA) according to the manufacturer’s instructions. After 48 h of treatment, 100 µL reagent was added per well, plates were shaken for 30 s, and then incubated for 1 h at room temperature before luminescence was recorded with a microplate reader.

### 2.8. Statistical Analysis

Data are presented as mean ± SD. Statistical analyses were performed using SAS software version 9.4 (SAS Institute Inc., Cary, NC, USA). Cell death rates were analyzed using linear mixed-effects models (PROC MIXED) to account for the hierarchical experimental structure. Culture condition (2D vs. 3D), treatment, and their interaction (culture × treatment) were specified as fixed effects, while biologically independent experiments were modelled as a random intercept to account for between-experiment variability. Restricted maximum likelihood (REML) estimation was used, and denominator degrees of freedom were calculated using the Kenward–Roger method. Overall effects were assessed using Type III tests of fixed effects. To further explore culture-dependent differences within individual treatments, slice tests were performed to compare 2D versus 3D cultures separately for each treatment. Drug interactions were analyzed for each drug pair using two-factor linear models (PROC MIXED) with the two agents and their interaction as fixed effects. The response (effect) additivity model served as the reference: the expected additive effect of a combination was defined as the sum of the single-agent effects relative to the vehicle control. Deviation from additivity was quantified as difference-in-differences (DiD) contrast, DiD = [E(A + B) − E(B)] − [E(A) − E(0)], where E denotes the model-based mean response under the respective treatment condition; this contrast is equivalent to the A × B interaction term and was reported with 95% confidence intervals. A non-significant DiD was interpreted as no evidence of deviation from additivity, whereas significant deviations were classified as greater-than-additive (synergistic) or less-than-additive (antagonistic) according to the direction of the treatment effect. Accordingly, a positive DiD indicates a greater-than-additive effect for specific cell death, but a less-than-additive effect for ATP levels. Model-based least-squares means (LSMeans) and associated standard errors were reported. All statistical tests were two-sided, and *p* values < 0.05 were considered statistically significant. The global culture × treatment interaction test was considered the primary test for culture-dependent treatment effects. Slice tests comparing 2D versus 3D cultures within individual treatments were performed only following a significant global interaction and were interpreted as exploratory, descriptive analyses; the corresponding *p*-values are reported without adjustment for multiple comparisons. Likewise, DiD contrasts were not adjusted for multiplicity. Bonferroni correction was applied to the pairwise comparisons of caspase-3/7 activity (four comparisons; significance threshold *p* < 0.0125). Differences between two independent groups were analyzed using a two-tailed Welch’s *t*-test.

## 3. Results

HCC treatment guidelines recommend stage-dependent strategies, from curative interventions to systemic therapies, including tyrosine kinase inhibitors and immune checkpoint inhibitors [2,14,20,53,54]. Sorafenib and regorafenib have long been standard tyrosine kinase inhibitors and remain important treatment options. NCT-503 has been shown to potentiate the antitumor activity of sorafenib and other compounds, described by the authors as synergistic [41,42], while the histone deacetylase inhibitor panobinostat is known to induce cell death [36,37,38]. Here, we investigated the effects of these compounds on the HCC cell line HepG2, both as single agents and in combination, and assessed whether responses differed between two-dimensional (2D) and three-dimensional (3D) culture systems.

### 3.1. Kinase Inhibition in Combination with Epigenetic Modulation or Metabolic Targeting Promotes Cell Death

**Figure 1:**
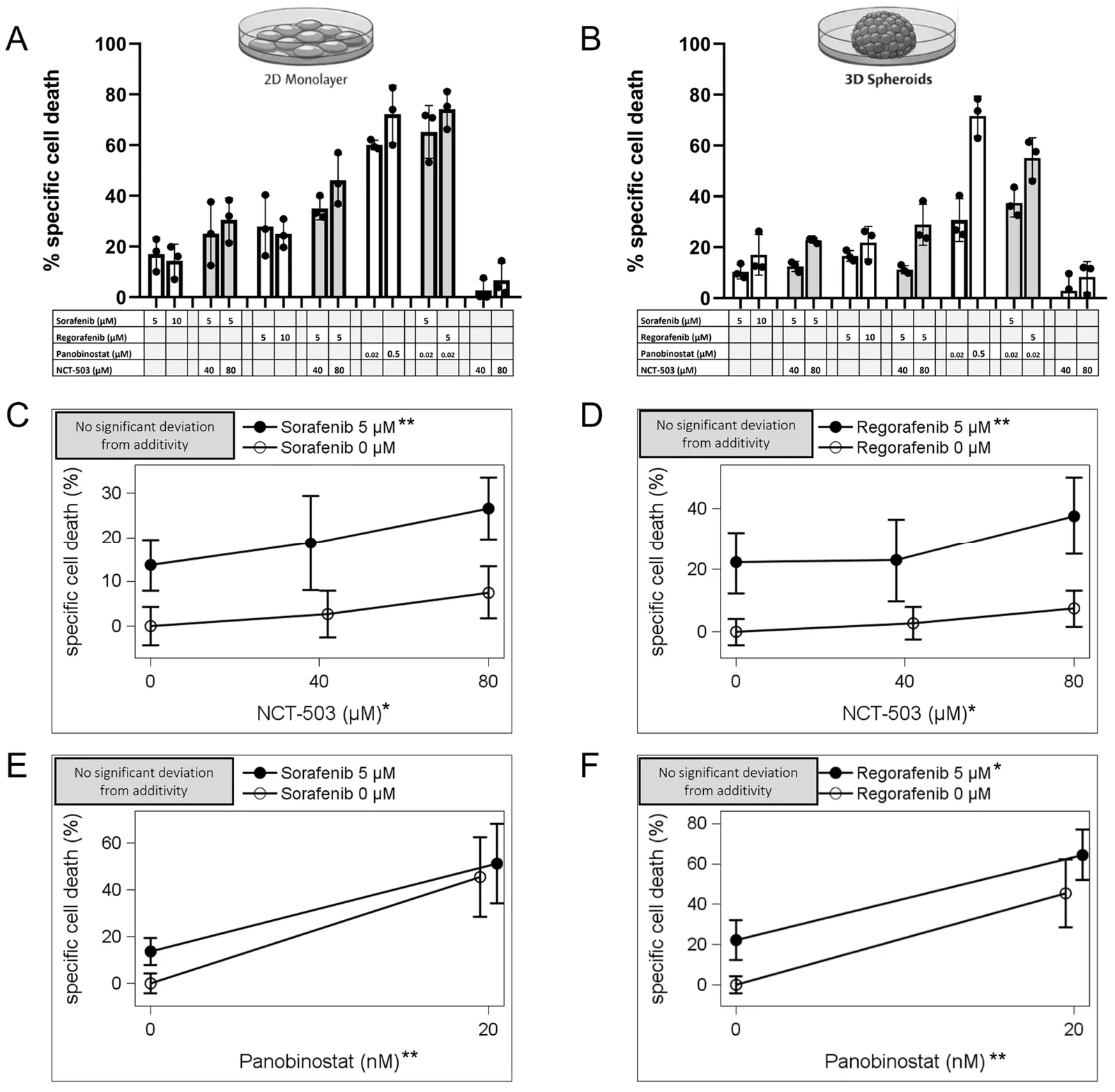
Induction of cell death in 2D- and 3D-cultured HepG2 cells upon treatment with kinase inhibitors and epigenetic or metabolic modulators. HepG2 cells were cultured under (**A**) 2D conditions or as (**B**) 3D spheroids and treated with sorafenib, regorafenib, NCT-503, and panobinostat or their combinations for 48 h. Specific cell death was determined by flow cytometry after Annexin V/DAPI staining (n = 3 independent biological experiments). Solvent-treated cells (corresponding concentration of DMSO) served as control. (**C**–**F**) Formal interaction and difference-in-differences (DiD) analyses were conducted to evaluate potential synergistic interactions between kinase inhibitors and epigenetic or metabolic modulators. Pooled specific cell death rates after treatment in 2D and 3D cultures were analyzed (n = 6). (**C**) * = Type III test NCT-503 effect: *p* = 0.032, ** = Type III Test sorafenib effect: *p* < 0.0001; sorafenib x NCT-503 interaction showed no significant deviation from additivity: *p* = 0.6177. (**D**) * = Type III test NCT-503 effect: *p* = 0.011, ** = Type III test regorafenib effect: *p* < 0.0001; regorafenib x NCT-503 interaction showed no significant deviation from additivity: *p* = 0.41. (**E**) ** = Type III test panobinostat effect: *p* < 0.0001; sorafenib x panobinostat interaction showed no significant deviation from additivity: *p* = 0.4562. (**F**) * = Type III test regorafenib effect: *p* = 0.0004, ** = Type III test panobinostat effect: *p* < 0.0001; regorafenib x panobinostat interaction showed no significant deviation from additivity: *p* = 0.7547.

To determine the proportion of cell death induced by the respective compounds in conventional 2D culture, HepG2 cells were treated for 48 h with sorafenib (5, 10 µM), regorafenib (5, 10 µM), NCT-503 (40, 80 µM), or panobinostat (0.02 µM, 0.5 µM), either as single agents or in combination. Cell death was subsequently assessed by Annexin V/DAPI staining followed by flow cytometric analysis (Figure 1A). Sorafenib induced specific cell death rates of 17% at 5 µM and 14.3% at 10 µM, whereas regorafenib resulted in 27.9% and 25.0% at 5 µM and 10 µM, respectively. NCT-503 alone induced only low levels of cell death (2.7% at 40 µM and 6.7% at 80 µM). However, a combination of sorafenib (5 µM) with NCT-503 increased cell death to 25.1% and 30.6% at 40 µM and 80 µM, respectively. Similar effects were observed for regorafenib combined with NCT-503, resulting in 35.0% and 46.2% cell death. The strongest effects were observed with the HDAC inhibitor panobinostat, which induced 60.1% cell death at 0.02 µM and 72.2% at 0.5 µM. However, combinations with sorafenib or regorafenib did not further significantly enhance these effects. Taken together, in 2D cultures, the kinase inhibitors sorafenib and regorafenib produced strong increases in specific cell death across all tested conditions. Panobinostat, and at higher doses, NCT-503, also induced cytotoxicity as single agents and further increased cell death when combined with kinase inhibition (Figure 1A).

We next evaluated whether these cell death-inducing effects of the compounds were preserved in 3D cell culture. After 24 h of 3D culture, stable and well-defined HepG2 spheroids had formed and were exposed to the same compounds and combinations as in 2D for 48 h. After 48 h, spheroids were dissociated, stained with Annexin V/DAPI, and analyzed by flow cytometry (Figure 1B). Sorafenib induced 10.4% and 17% cell death at 5 µM and 10 µM, respectively, while regorafenib resulted in 16.6% and 21.8% cell death. NCT-503 single-agent treatment showed the lowest effects, with 2.9% and 8.3% cell death at 40 µM and 80 µM. A combination of sorafenib (5 µM) with 40 µM NCT-503 did not increase cell death compared with sorafenib alone, whereas 80 µM NCT-503 enhanced cell death. In contrast, no relevant increase was observed for combinations of regorafenib with NCT-503. As in 2D culture, panobinostat-induced cell death in spheroids was not further enhanced by combination with either sorafenib or regorafenib (Figure 1B). Thus, in 3D cultures, all compounds remained biologically active, with each single-agent treatment increasing cell death. Combination treatments generally further enhanced cell death, except for NCT-503 combined with regorafenib, which showed no additional effect compared with regorafenib alone.

### 3.2. Kinase Inhibitor-Based Combinations with Epigenetic or Metabolic Targeting Result in Enhanced Cell Death Without Evidence of Synergy

To assess potential synergistic interactions between kinase inhibitors and epigenetic or metabolic modulators, we performed formal interaction and difference-in-differences (DiD) analyses for sorafenib or regorafenib in combination with panobinostat or NCT-503 in both 2D and 3D culture. Because separate DiD analyses in 2D and 3D cultures yielded no deviation from additivity in both systems (Figures S1 and S2), cell death data from both culture formats were pooled for the combined analysis to increase statistical power.

A two-factor linear mixed-effects model including sorafenib, NCT-503, and their interaction revealed a strong main effect of sorafenib on cell death (Type III *p* < 0.0001) and a significant main effect of NCT-503 (Type III *p* = 0.0032), indicating that both compounds modulate cell death across the combined dataset (Figure 1C). In contrast, the global sorafenib × NCT-503 interaction term was not significant (*p* = 0.6177), providing no evidence for a consistent non-additive (synergistic or antagonistic) interaction across dose combinations. To formally test non-additivity at each NCT dose, DiD contrasts compared the NCT-associated change under sorafenib (5 µM) versus no sorafenib. These analyses showed no significant deviation from additivity for either dose: DiD (NCT 40 vs. 0) = +2.28 (95% CI −8.97 to 13.53; *p* = 0.682) and DiD (NCT 80 vs. 0) = +5.43 (95% CI −5.82 to 16.68; *p* = 0.332).

Regarding regorafenib (0 vs. 5 µM), NCT-503 (0, 40, 80 µM), and their interaction, the two-factor linear mixed-effects model revealed highly significant main effects for both regorafenib (*p* < 0.0001) and NCT-503 (*p* = 0.011) (Figure 1D). The global interaction term between regorafenib and NCT-503 was not statistically significant (Type III test: *p* = 0.41), indicating no evidence for a consistent non-additive interaction across doses. This finding was corroborated by DiD analyses, which showed no significant deviation from additivity for either NCT-503 concentration (40 µM: DiD = −1.9 (95% CI −17.33 to 13.53; *p* = 0.80); 80 µM: DiD = +7.8 (95% CI −7.63 to 23.23; *p* = 0.31).

Panobinostat treatment resulted in a pronounced increase in cell death across conditions (Figure 1E). In the absence of sorafenib, panobinostat at 0.02 µM significantly increased apoptotic cell death compared with the untreated controls (*p* < 0.0001). Sorafenib alone induced a smaller increase in cell death compared with the untreated controls but the main effect of sorafenib did not reach significance (*p* = 0.0692). Formal testing for non-additive drug interactions revealed no evidence for a non-additive interaction between sorafenib and panobinostat (sorafenib × panobinostat, *p* = 0.4562), indicating that the combined effect did not differ from the sum of the individual drug effects. Consistently, the DiD contrast comparing the panobinostat effect in the presence versus absence of sorafenib was negative but not significant (DiD estimate −7.77, 95% CI −29.1 to 13.6, *p* = 0.456). Taken together, these results suggest that panobinostat robustly induces cell death irrespective of sorafenib exposure, while the combination showed no evidence of synergy.

Both regorafenib and panobinostat exerted strong and highly significant main effects on cell death rates. Regorafenib treatment significantly increased cell death compared with untreated controls (*p* = 0.0004), and panobinostat induced a pronounced increase in specific cell death across conditions (*p* < 0.0001). The formal interaction term between regorafenib and panobinostat did not reach statistical significance (*p* = 0.7547), indicating no evidence for a non-additive interaction. Consistently, the DiD contrast comparing the panobinostat effect in the presence versus absence of regorafenib was not significant (DiD = −3.07, 95% CI −23.29 to 17.15, *p* = 0.7547). Taken together, these results show that regorafenib and panobinostat independently promote apoptotic cell death, but their combined effect showed no significant deviation from additivity, without detectable synergistic or antagonistic interaction.

Collectively, these data indicate that each drug individually contributes to cell death induction, with no significant deviation from additivity observed for the tested combinations.

### 3.3. Kinase Inhibition and Epigenetic/Metabolic Targeting Converge on the Bioenergetic State of HepG2 Cells

**Figure 2:**
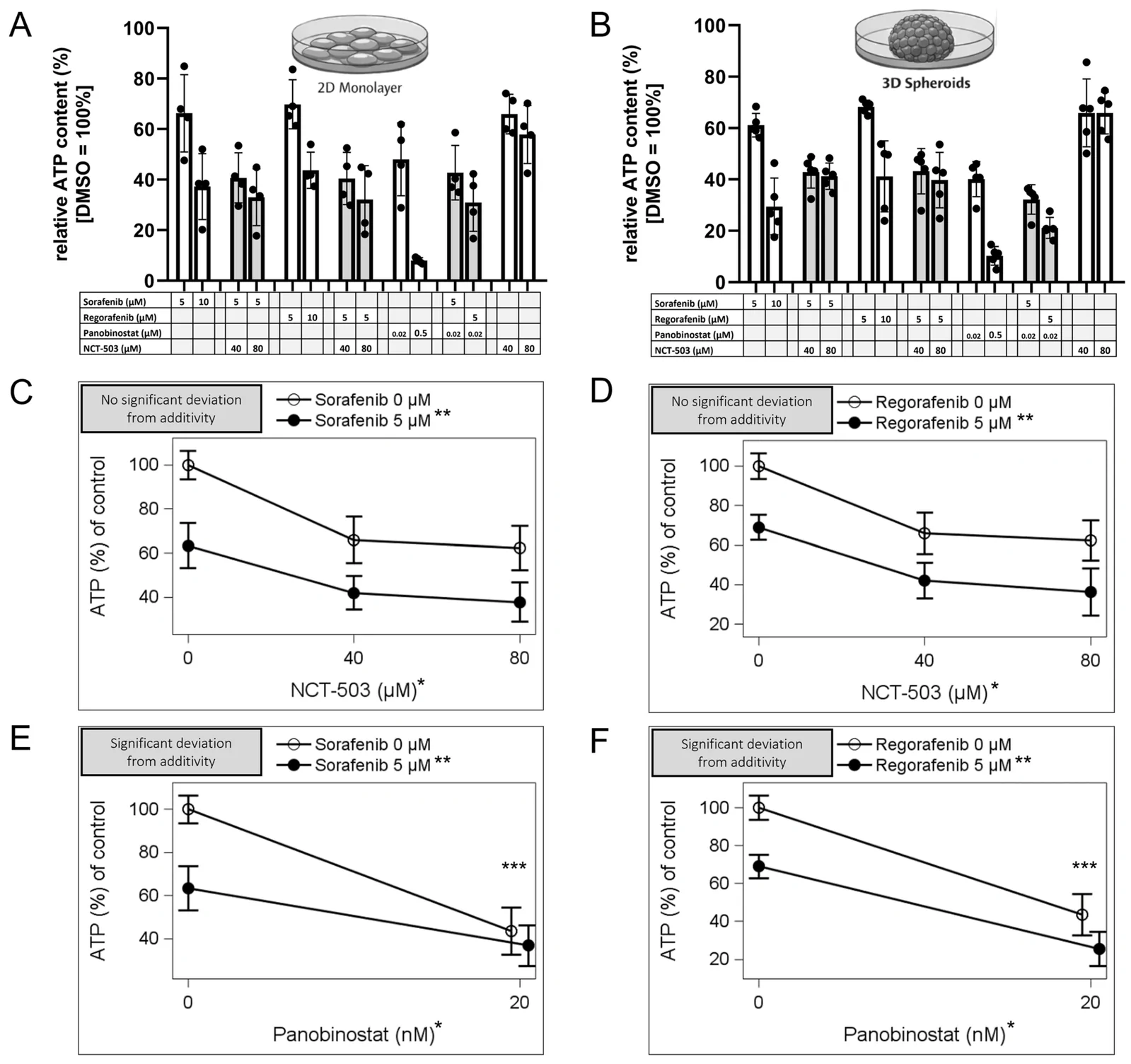
Combination of kinase inhibitors with epigenetic or metabolic agents results in ATP depletion. HepG2 cells were cultured under (**A**) 2D conditions or as (**B**) 3D spheroids and treated with sorafenib, regorafenib, NCT-503, and panobinostat or their combinations for 48 h. Cellular energy levels were determined using an ATP assay (n = 4–5 independent biological experiments). Solvent-treated cells (corresponding concentration of DMSO) served as control. (**C**–**F**) Formal interaction and difference-in-differences (DiD) analyses were performed to assess potential synergistic interactions between kinase inhibitors and epigenetic or metabolic modulators. Pooled ATP data after treatment in 2D and 3D cultures were analyzed (n = 9). (**C**) * = Type III test NCT-503 effect: *p* < 0.0001, ** = Type III test sorafenib effect: *p* < 0.0001; sorafenib x NCT-503 interaction showed no significant deviation from additivity: *p* = 0.0753. (**D**) * = Type III test NCT-503 effect: *p* < 0.0001, ** = Type III test regorafenib effect: *p* < 0.0001; regorafenib x NCT-503 interaction showed no significant deviation from additivity: *p* = 0.5041. (**E**) * = Type III test panobinostat effect: *p* < 0.0001, ** = Type III test sorafenib effect: *p* < 0.0001; *** = sorafenib x panobinostat interaction showed significant deviation from additivity: *p* < 0.0001. (**F**) * = Type III test panobinostat effect: *p* < 0.0001, ** = Type III test regorafenib effect: *p* < 0.0001; *** = regorafenib x panobinostat interaction showed significant deviation from additivity: *p* = 0.0287.

Cellular energy status is a critical determinant of cell death induction and influences which death pathway is activated. We therefore assessed intracellular ATP levels in HepG2 cells as a function of culture conditions (2D vs. 3D) and drug treatment. HepG2 cells were initially cultured under conventional 2D conditions and treated with sorafenib, regorafenib, NCT-503, or panobinostat. After 48 h of treatment, cellular ATP content was quantified using a luminescence-based ATP assay. All compounds reduced cellular ATP levels in a dose-dependent manner (Figure 2A). Sorafenib decreased the ATP content to ~70% of the control at 5 µM and to 37.3% at 10 µM. Regorafenib produced a comparable decline to 69.8% (5 µM) and 43.7% (10 µM). NCT-503 lowered the ATP levels to 66% at 40 µM and 57.9% at 80 µM. Notably, this pronounced metabolic effect contrasted with the relatively low cell death rates observed for NCT-503 alone (2.7% and 6.7%, respectively; Figure 1A). In combination settings, 80 µM NCT-503 significantly enhanced the ATP-reducing effect of sorafenib, while both 40 µM and 80 µM NCT-503 potentiated the effect of regorafenib. Panobinostat caused the strongest ATP depletion, reducing the levels to 48% at 0.02 µM and 8% at 0.5 µM, with no additional effects upon co-treatment with sorafenib or regorafenib.

ATP levels were subsequently measured in 3D HepG2 spheroids after treatment with sorafenib, regorafenib, NCT-503, or panobinostat. Spheroids were incubated with the respective compounds for 48 h and ATP content was quantified (Figure 2B). Overall, the effects on spheroid energy metabolism closely mirrored those observed in 2D culture. Sorafenib and regorafenib induced a significant reduction in ATP levels when concentrations were increased from 5 µM to 10 µM. NCT-503-treated samples maintained a comparable energy level (~65.9%) to 2D conditions at 40 µM, but no clear dose dependency at 80 µM was detected. Similar to 2D, combining NCT-503 with either sorafenib or regorafenib resulted in a significant additional decrease in ATP content at the low dosages of sorafenib and regorafenib. Treatment with 0.02 µM panobinostat caused a modest ATP reduction in combination with sorafenib, whereas co-treatment with regorafenib led to a significant decrease relative to panobinostat alone (Figure 2B).

Together, these data demonstrate that all tested agents markedly impaired cellular energy status in both 2D- and 3D-cultured HepG2 cells.

### 3.4. Kinase Inhibitor-Based Drug Combinations with Epigenetic or Metabolic Modulators Reduce Cellular Energy Levels

To determine whether the observed changes in cellular energy metabolism followed a similar interaction pattern, we applied the same interaction and DiD framework to ATP measurements obtained after drug treatment. As the response patterns were comparable between 2D and 3D models, and because separate DiD analyses in 2D and 3D cultures yielded similar interaction patterns in both systems (Figures S3 and S4), ATP data from both culture formats were pooled for the combined analysis to increase statistical power (Figure 2C–F).

Sorafenib and NCT-503 both strongly reduced ATP in a dose-dependent manner (Type III: sorafenib *p* < 0.0001; NCT-503 *p* < 0.0001). In the absence of sorafenib, NCT-503 decreased ATP by 34.1% (40 µM) and 37.7% (80 µM), while in combination with sorafenib, reductions were 27.0% and 32.6%, respectively. The sorafenib × NCT-503 interaction was not significant (*p* = 0.075). However, targeted DiD analyses revealed evidence for non-additive effects. At 40 µM NCT-503, a significant positive interaction was observed (DiD = +12.6%, 95% CI 0.4–24.7, *p* = 0.044), indicating that the reduction in ATP levels was attenuated in the presence of sorafenib compared with the expected additive effect. At 80 µM NCT-503, a similar trend was present but did not reach statistical significance (DiD = +12.0%, *p* = 0.054) (Figure 2C).

Regorafenib and NCT-503 showed a similar pattern: each agent significantly decreased ATP (regorafenib: *p* < 0.0001; NCT-503: *p* < 0.0001), with no significant deviation from additivity in combination (DiD: +7–5%, n.s.) (Figure 2D).

Sorafenib and panobinostat each strongly reduced ATP levels (Type III: sorafenib *p* < 0.0001; panobinostat *p* < 0.0001). However, a significant sorafenib × panobinostat interaction was detected (*p* < 0.0001), with panobinostat-induced ATP depletion attenuated in the presence of sorafenib (0.02 µM pano: −56.4% alone vs. −26.5% with sorafenib; DiD = +29.8%, 95% CI 16.99–42.66; *p* < 0.0001), indicating a significant deviation from the expected additive effect (Figure 2E).

Regorafenib and panobinostat also showed significant main effects (regorafenib: *p* < 0.0001; panobinostat: *p* < 0.0001), and the combination produced a less-than-additive effect (0.02 µM panobinostat: −56.4% alone vs. −43.5% with regorafenib; interaction, *p* = 0.0287; DiD = +12.9%, 95% CI 1.4–24.3; *p* = 0.0287) (Figure 2F).

Taken together, all tested kinase inhibitors, metabolic, and epigenetic modulators markedly reduced cellular ATP levels. While the overall interaction analysis for combinations with NCT-503 did not indicate a significant deviation from additivity, an individual significant DiD contrast was observed for sorafenib and 40 µM NCT-503, which is considered exploratory. In contrast, an attenuated response was observed for panobinostat when combined with either sorafenib or regorafenib, indicating that combined energy depletion does not always scale linearly with single-agent effects.

### 3.5. Distinct Mechanisms of Cell Death Induced by Kinase Inhibitor Combinations with Epigenetic or Metabolic Modulators

**Figure 3:**
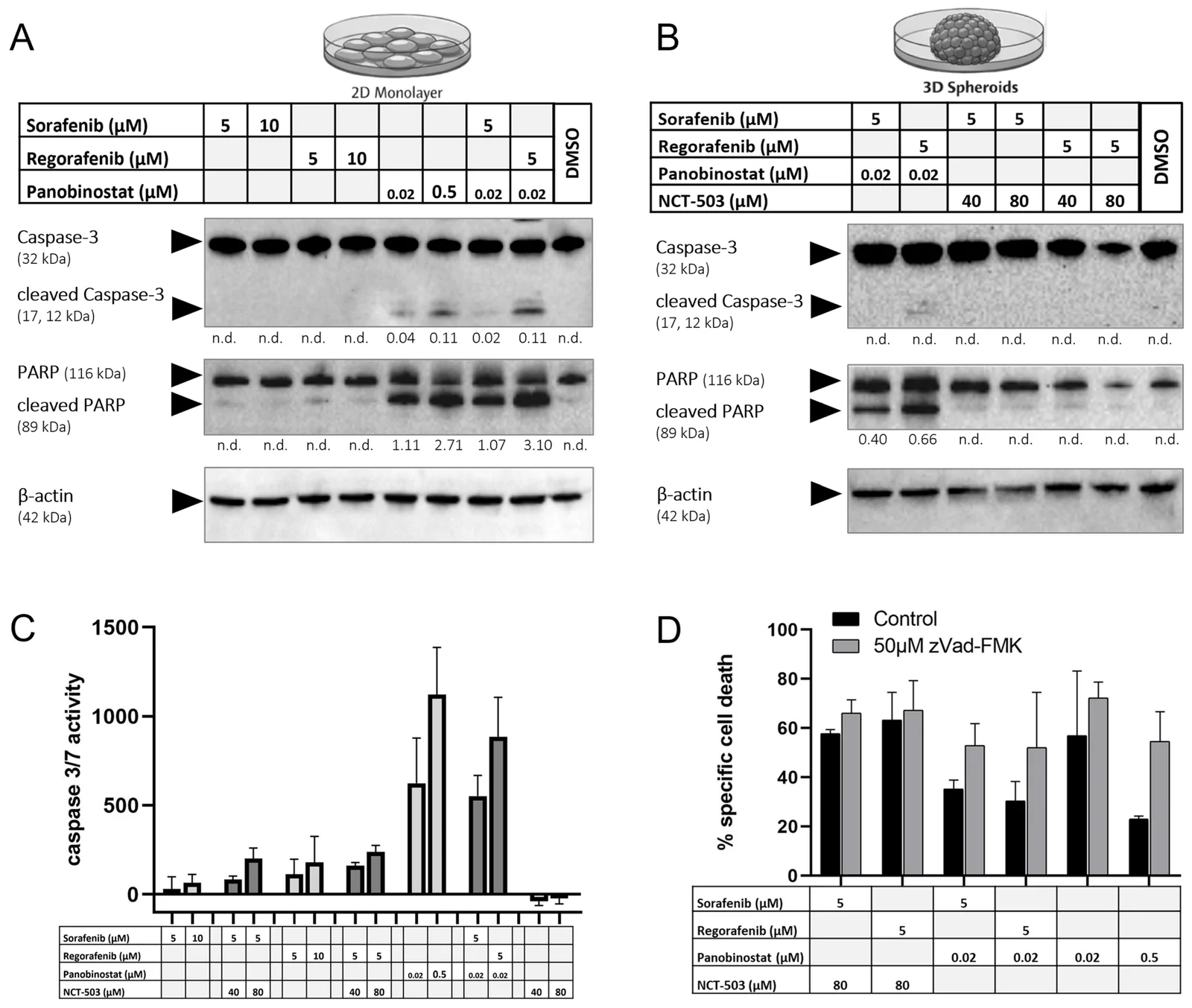
Distinct apoptotic and caspase-independent effects of kinase inhibitor combinations with epigenetic or metabolic agents. HepG2 cells were incubated in (**A**) 2D monolayer culture or as (**B**) 3D spheroids for 24 h in the presence of the indicated compounds. Caspase-3 and PARP were analyzed by Western blot. β-actin served as a loading control. Representative Western blots from one of three independent experiments are shown. Densitometric analysis of the representative Western blots is presented as the ratio of cleaved to full-length PARP and caspase-3, respectively (n.d. = not detected). (**A**) Panobinostat and its combinations induced apoptotic signaling, while (**B**) NCT-503-dependent cell death was independent of canonical caspase-mediated apoptosis. (**C**) HepG2 cells were incubated in 2D culture for 48 h with the indicated compounds (sorafenib, regorafenib, NCT-503, and panobinostat) and their combinations. Caspase-3 and -7 activity was measured using a functional luminescence-based assay. DMSO-treated cells served as internal control (n = 4 independent biological experiments). (**D**) Cell death induced by NCT-503 combinations was only minimally reduced by the caspase inhibitor zVAD-FMK. HepG2 cells were cultured in 2D and incubated for 48 h with the indicated compounds in the presence or absence of 50 µM zVAD-FMK. Cells were stained with Annexin V/DAPI and analyzed by flow cytometry. The proportion of viable cells is shown. Data are presented as mean ± SD (n = 3 independent biological experiments).

To determine whether the observed cell death involved apoptosis, HepG2 cells cultured in 2D and 3D were treated with sorafenib, regorafenib, NCT-503, or panobinostat for 24 h, and cell lysates were subsequently analyzed by Western blot (Figure 3).

Analysis of caspase-3 activation revealed clear cleavage of the executioner caspase following treatment with panobinostat in 2D culture and following panobinostat combinations in 3D cultures, confirming activation of the canonical apoptotic pathway. The combination with sorafenib did not further enhance caspase-3 processing, whereas co-treatment with regorafenib modestly increased panobinostat-induced cleavage, especially in 2D (Figure 3A) and a weaker effect in 3D (Figure 3B). In contrast, NCT-503 applied in combination with kinase inhibitors did not induce detectable caspase-3 activation (Figure 3B).

Consistent with these findings, cleavage of poly(ADP-ribose) polymerase (PARP), a downstream substrate of activated caspase-3, was observed predominantly in the presence of panobinostat. In 2D cultures, pronounced PARP processing occurred only after panobinostat treatment, while sorafenib or regorafenib alone induced only minor effects (Figure 3A). A similar pattern was detected in 3D spheroids, where combination treatments with panobinostat also induced PARP cleavage, although to a lower extent than that observed in 2D, which is consistent with the attenuated cell death response. In contrast, combinations of NCT-503 and kinase inhibitors produced weaker PARP processing (Figure 3B).

To further assess caspase activation, a functional caspase-3/7 activity assay was performed in 2D-cultured HepG2 cells treated with sorafenib, regorafenib, NCT-503, or panobinostat for 48 h. Consistent with the Western blot data, robust caspase-3/7 activation was detected primarily in panobinostat-treated samples (Figure 3C). This finding further demonstrated that the assay was responsive under our experimental conditions. Sorafenib and regorafenib induced only weak increases in caspase activity, which were modestly enhanced by co-treatment with NCT-503, reaching significance for the combination of sorafenib with 80 µM NCT-503 (*p* = 0.009, Welch’s *t*-test, after Bonferroni correction for four comparisons, results were considered statistically significant at *p* < 0.0125). Owing to its higher sensitivity, this assay indicates that caspase activation is primarily associated with panobinostat-containing regimens, whereas kinase inhibitors and NCT-503 induced comparatively limited caspase activation.

Despite modest caspase activation observed with sorafenib, regorafenib, NCT-503, and their combinations, flow cytometry revealed substantial specific cell death rates of up to 47% (Figure 1), suggesting the involvement of additional, non-apoptotic mechanisms. To further dissect the mode of cell death, HepG2 cells were treated in 2D culture with sorafenib, regorafenib, NCT-503, or panobinostat in the presence or absence of the pan-caspase inhibitor zVAD-FMK, followed by flow cytometric analysis after 48 h. Consistent with earlier findings demonstrating robust canonical apoptotic signaling upon panobinostat treatment, zVAD-FMK markedly and significantly reduced panobinostat-induced cell death. The fraction of viable cells increased from 22.97% to 54.64% (*p* = 0.03, Welch’s *t*-test). Similar caspase-dependent protection was observed in combinations of panobinostat with sorafenib or regorafenib (Figure 3D). In contrast, zVAD-FMK did not confer comparable protection in cells treated with NCT-503 alone or in combination with kinase inhibitors. Viability remained largely unchanged (63.31% without vs. 67.32% with zVAD-FMK). Likewise, cell death induced by the combination of NCT-503 and sorafenib was unaffected by caspase inhibition (Figure 3D). These findings align with the weak caspase activation and limited PARP cleavage observed under these conditions and indicate that NCT-503-based treatments predominantly trigger pathways independent of canonical caspase activation.

Taken together, these data suggest that panobinostat-containing regimens induce largely caspase-dependent apoptosis, whereas NCT-503-containing combinations induce cell death independent of canonical caspase-mediated apoptosis in 2D cultures. Although the 3D findings were consistent with this interpretation, the specific cell death pathway underlying the NCT-503-associated response remains to be determined.

### 3.6. 3D Culture Confers Resistance to Treatment-Induced Cell Death Without Altering ATP Levels

**Figure 4:**
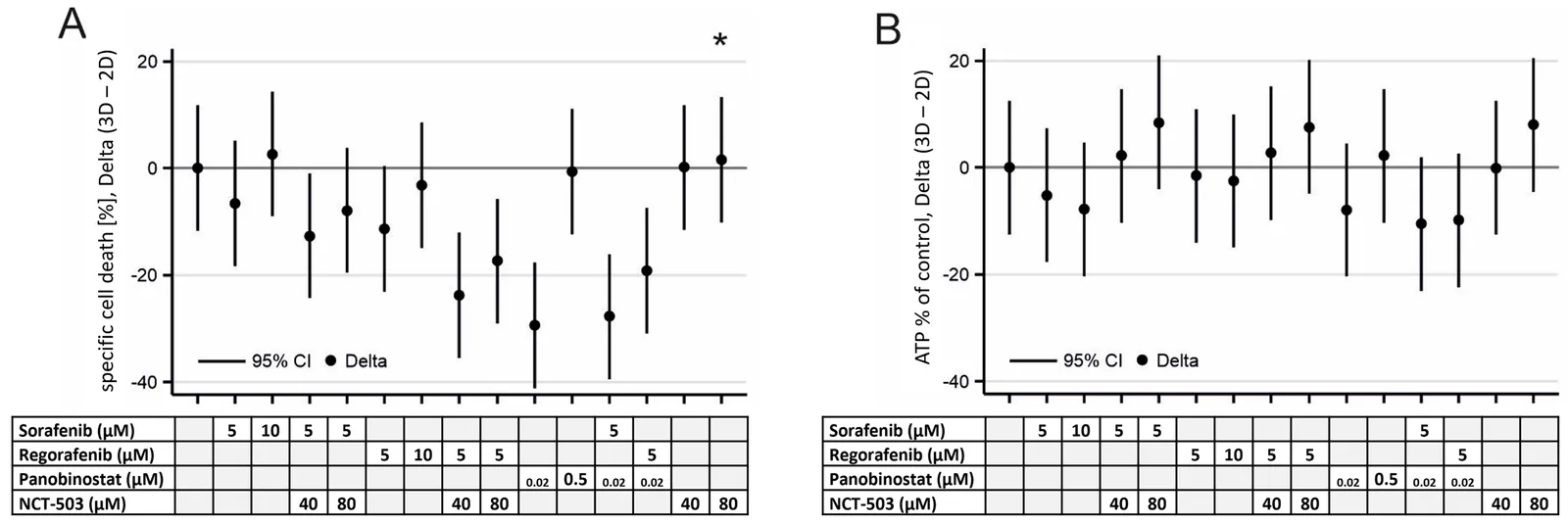
3D culture reduces treatment-induced cell death independently of changes in cellular energy status. Differences (delta = 3D − 2D) in (**A**) specific cell death and (**B**) cellular ATP levels were calculated for HepG2 cells under each treatment condition. Data are presented as delta values with 95% confidence intervals. Statistical analyses were performed using a mixed-effects model including culture (2D vs. 3D), treatment, and their interaction. * = Type III test culture x treatment interaction: *p* < 0.0001 for specific cell death; no significant culture x treatment interaction for ATP: *p* = 0.40.

To assess whether culture conditions influence cell death independently of treatment effects, cell death rates were analyzed using linear mixed-effects modelling. Culture (2D vs. 3D), treatment, and their interaction were included as fixed effects, with biologically independent experiments modelled as a random intercept. A significant culture × treatment interaction was observed (Type III test, *p* = 0.0001), indicating that the effect of culture conditions on specific cell death depended on the treatment applied. Post hoc slice tests comparing 2D versus 3D cultures within each treatment revealed significantly higher specific cell death rates in 2D cultures for several treatments, most prominently for panobinostat-containing and regorafenib-based combination treatments. The nominally significant difference observed for sorafenib combined with 40 µM NCT-503 (*p* = 0.0349) should be interpreted with caution given the number of treatment-specific comparisons. In contrast, no treatment showed significantly higher cell death rates in 3D cultures, and for several treatments, no culture-dependent difference was detected (Table 1, Figure 4A). These findings are consistent with 3D culture conditions selectively attenuating specific cell death responses to distinct drugs and drug combinations rather than exerting a uniform effect across all treatments.

In contrast to specific cell death, ATP levels were not significantly affected by the culture conditions. In a mixed-effects model including culture (2D vs. 3D), treatment, and their interaction, no main effect of culture (*p* = 0.66) and no culture-by-treatment interaction (*p* = 0.40) were observed (Figure 4B). Slice analyses confirmed the absence of significant ATP differences between 2D and 3D cultures across all treatment conditions. These results indicate that treatment-induced changes in ATP are largely culture-independent, highlighting a dissociation between metabolic activity and cell death responses.

Taken together, these data suggest that 3D cultures of HepG2 cells exhibited reduced drug-induced cell death despite comparable energy depletion compared with 2D cultures.

## 4. Discussion

Despite current guideline-recommended systemic therapies, which primarily include immune checkpoint inhibitor combinations and tyrosine kinase inhibitors, durable control of HCC is often challenging due to intrinsic and acquired resistance mechanisms [14,18,55]. Since the introduction of sorafenib in 2007, systemic therapy for advanced HCC has largely been based on tyrosine kinase inhibitors. Although additional agents such as lenvatinib and regorafenib expanded treatment options, clinical progress remained limited for many years [55,56,57,58,59,60]. More recently, immune checkpoint inhibitor-based combinations, including atezolizumab plus bevacizumab, tremelimumab plus durvalumab, camrelizumab plus rivoceranib, ipilimumab plus nivolumab and sintilimab plus bevacizumab biosimilar (IBI305), have improved clinical outcomes [14,17,18,19,21,22,23,55,57,58,59,60]. However, therapeutic responses remain heterogeneous, and resistance frequently emerges. In line with these limitations, resistance to kinase-targeted therapies is increasingly attributed to adaptive mechanisms that sustain tumor survival despite pathway inhibition, as illustrated by a recent study implicating leucine-tRNA synthetase-mediated regulation of the PI3K/AKT/mTOR pathway in HCC cell proliferation and survival [61]. In particular, epigenetic reprogramming and metabolic rewiring can compensate for suppressed kinase signaling and maintain proliferative capacity. Accordingly, combining tyrosine kinase inhibitors with epigenetic or metabolic modulators has emerged as a promising strategy to overcome such adaptive resistance mechanisms [62,63,64]. By simultaneously targeting signaling pathways and cellular regulatory programs, these combinatorial approaches aim to exploit complementary vulnerabilities and improve therapeutic efficacy. In addition, substantial progress has been made in the development of physiologically relevant 3D culture systems. These models increasingly enable more predictive in vitro drug-testing approaches by better recapitulating key aspects of tumor architecture and microenvironment. In this study, we therefore evaluated tyrosine kinase inhibitors in combination with epigenetic and metabolic modulators in both 2D cultures and 3D spheroid models to assess treatment efficacy and resistance under more physiologically relevant conditions.

We evaluated the effects of the clinically established drugs sorafenib, regorafenib, and panobinostat, alongside the experimental PHGDH inhibitor NCT-503, in HepG2 cells. Both single-agent and combination treatments were tested across multiple concentrations. While all agents increased cell death, combinations predominantly showed no deviation from additivity, indicating enhanced cytotoxicity without evidence of synergy.

HDAC inhibitors, like panobinostat, represent a promising class of therapeutics across various malignancies. In HCC, HDAC5 is frequently overexpressed in tumor tissue and associated with increased cell proliferation [65], while high levels of HDAC1 and HDAC2 correlate with poor patient prognosis [66,67]. Overexpression of class I HDACs has also been observed in other cancers, including prostate, esophageal, and non-small-cell lung carcinoma [68], further highlighting their potential as therapeutic targets.

Panobinostat single-agent treatment induced dose-dependent cell death and reduced viability in HepG2 cells in both 2D and 3D cultures. The effect was caspase-dependent and mediated by apoptosis, as shown by caspase-3 and PARP cleavage, increased caspase-3/7 activity, and its inhibition by zVAD-FMK. These findings are consistent with previous reports showing that panobinostat induces apoptosis and reduces cellular proliferation across various cancer cell types [38,69,70,71]. Due to their broad biological effects on gene regulation, apoptosis, cell cycle progression, immune responses, and resistance pathways, HDAC inhibitors represent promising agents for combination therapies. Numerous preclinical and clinical studies have shown that their combination with other therapeutics can induce synergistic effects and overcome resistance mechanisms [72,73,74]. Despite promising preclinical evidence of synergistic effects between HDAC inhibitors and other therapies, including kinase inhibitors [75,76,77,78,79,80], their clinical use remains limited. A phase I/II trial in advanced HCC showed no significant benefit of combining the HDAC inhibitor resminostat with sorafenib compared with sorafenib monotherapy [81]. In addition, clinical application is constrained by treatment-related toxicities, including hematological adverse events (anemia, neutropenia, thrombocytopenia), and other side effects such as fatigue, diarrhea, and nausea observed with panobinostat [82,83]. Nevertheless, panobinostat combined with sorafenib enhances the reduction in cell viability and proliferation and increases apoptosis compared with monotherapy [75]. Consistently, our group demonstrated synergistic apoptosis induction for vorinostat and sorafenib in cutaneous T-cell lymphoma [84]. To date, the combination of HDAC inhibitors and regorafenib has rarely been investigated in the literature [85]. In the present study, combinations of panobinostat with sorafenib or regorafenib enhanced cell death and reduced cellular ATP levels in both 2D and 3D cultures; however, they did not significantly exceed the expected additive effect. Of note, the less-than-additive deviations observed for some combinations in the ATP analyses, particularly those containing panobinostat, should be interpreted with caution: because single-agent effects on ATP levels were large and the endpoint was bounded, the effect-additivity model predicted near-complete ATP depletion for these combinations, and the observed deviations may therefore reflect saturation of the ATP response rather than pharmacological antagonism. Apoptosis was identified as the underlying mechanism, as indicated by caspase-3 and PARP cleavage.

Building on previous findings by Wei et al. [41], we also investigated combinations of the PHGDH inhibitor NCT-503 with sorafenib and regorafenib in HepG2 cells, which additively increased cell death and reduced viability in both 2D and 3D cultures. Consistent with earlier reports showing that PHGDH inhibition disrupts redox homeostasis and induces ferroptosis through impaired NADPH and glutathione production [42], combination treatment in our study markedly enhanced cytotoxicity. In contrast, NCT-503 single-agent treatment did not significantly increase cell death but caused a pronounced reduction in cellular ATP levels, suggesting that PHGDH inhibition primarily affects cellular energy homeostasis rather than directly triggering cell death. Several studies indicate that PHGDH inhibition sensitizes tumor cells to additional therapies. NCT-503 enhanced the efficacy of tyrosine kinase inhibitors such as sorafenib, regorafenib, and lenvatinib in HCC models [41] and showed formally synergistic effects (CI < 1 or SynergyFinder-confirmed) with the glycolytic inhibitor PKM2-IN-1 in NSCLC [43] and with mTOR/PI3K-pathway modulators in osteosarcoma [86]. Potentiation of proteasome inhibitor activity (bortezomib) has also been reported in multiple myeloma [87]. As noted above, PHGDH inhibition increases oxidative stress and disrupts cellular redox homeostasis [42], which is associated with mitochondrial dysfunction and reduced ATP production [88,89]. Accordingly, the ATP depletion observed in our model likely reflects oxidative stress induced by PHGDH inhibition. While insufficient to trigger substantial cell death alone, NCT-503 markedly reduced the cellular ATP levels, whereas its combination with sorafenib or regorafenib increased cell death. However, whether this metabolic perturbation contributes to the enhanced cytotoxicity of these combinations in our model remains to be established. Moreover, the extent and nature of drug interactions may depend on the experimental model, treatment conditions, and method used to define drug interactions. In the present study, the tested combinations showed no significant deviation from the expected additive effect under the experimental conditions examined.

Mechanistically, the weak activation of caspase-3, limited PARP cleavage, and the lack of inhibition by zVAD-FMK suggest that canonical caspase-mediated apoptosis is not the primary mechanism of NCT-503-induced cell death, at least in 2D cultures. Consistent with this, Wang et al. reported ferroptosis as the underlying mechanism in NCT-503/enzalutamide combination treatment [42]. While our data do not clarify whether ferroptosis also contributes to the effects observed with sorafenib or regorafenib, the combination of the PHGDH inhibitor NCT-503 with these tyrosine kinase inhibitors may represent a promising therapeutic strategy for HCC, although further preclinical and clinical studies are required. The relatively high NCT-503 concentrations used in this study, particularly 80 µM, warrant the consideration of potential off-target effects. Arlt et al. reported PHGDH-independent alterations in glucose carbon flux following NCT-503 treatment, indicating that not all metabolic effects may be attributable to PHGDH inhibition [90]. Thus, off-target effects cannot be excluded.

To assess whether culture conditions affect drug response, HepG2 cells were treated with sorafenib, regorafenib, panobinostat, and NCT-503 in 2D and 3D cultures, using both single agents and combinations. All treatments increased cell death, with higher cytotoxicity observed in 2D. At first glance, this reflects greater drug accessibility in 2D, whereas 3D spheroids impose structural barriers, including cell–cell interactions, nutrient and oxygen gradients, and limited diffusion, creating a more physiologically relevant yet complex model system [91,92,93,94,95]. However, only minor treatment-dependent differences between 2D and 3D cultures were observed with regard to cellular energy levels. If the effects were solely attributable to differences in drug accessibility, a comparable pattern would also be expected in ATP levels. Instead, the discrepancy in cell death susceptibility between 2D and 3D cultures suggests that the 3D microenvironment may exert a protective function against certain drug-induced cell death mechanisms. These observations are consistent with previous reports demonstrating reduced drug sensitivity in 3D cultures compared with 2D models. Imamura et al. described increased drug resistance and reduced PARP cleavage following paclitaxel treatment in breast cancer spheroids, indicating diminished apoptotic responses in 3D systems [96]. Importantly, this effect depended on spheroid architecture, as loosely organized spheroids displayed drug responses comparable to 2D cultures. Similar findings were reported in breast and colorectal cancer models, where 3D cultures showed increased resistance to agents such as neratinib, docetaxel, 5-fluorouracil, and cisplatin, although drug- and cell line-specific differences were observed [97,98]. Moreover, these differential effects of cell death resistance were not only observed in response to pharmacological agents, but also to radiotherapy, as spheroid models have been shown to exhibit increased resistance compared with 2D cultures [98,99]. Building on these observations of increased resistance in 3D spheroids, recent studies indicate that this phenomenon is not solely due to limited drug penetration or structural barriers but also reflects underlying alterations in cellular signaling. Riedl et al. reported that 3D cultures exhibit reduced activity of key signaling pathways, including Akt and mTOR, compared with 2D models, potentially contributing to their increased drug resistance [100]. Thus, elevated resistance in 3D systems arises not only from physical barriers but also from complex molecular and cellular mechanisms protecting from drug-induced cell death.

In recent years, several 3D models of HCC have been established [101]. Mixed spheroids combining hepatoma cells with hepatic stellate cells have been shown to closely recapitulate the tumor-associated microenvironment, making them particularly suitable for studying stroma-dependent resistance mechanisms and evaluating novel therapeutic strategies [102,103]. HCC spheroids are also valuable for investigating potential cancer stem cell markers as targets for anti-stem cell therapies [101,104,105].

An increased drug tolerance observed in 3D HepG2 cultures despite comparable reductions in cellular ATP levels suggests that the 3D context may modulate the relationship between cellular energy homeostasis and drug-induced cell death, and that factors beyond the measured metabolic response contribute to the differential drug sensitivity. Other factors may also contribute to the differential response between 2D and 3D cultures. Although HepG2 cells express wild-type p53, potential differences in p53 pathway activity between culture conditions were not assessed. As p53 can modulate apoptotic responses and sensitivity to anticancer therapy [106,107], differences in p53 activity may contribute to the distinct treatment responses observed between 2D and 3D culture conditions. The use of both 2D and 3D HepG2 models provides complementary information on treatment responses across different culture contexts. However, the exclusive use of a single HCC cell line limits the generalizability of our findings. In addition, 3D experiments were performed using homotypic HepG2 spheroids and therefore do not recapitulate the cellular complexity of multicellular tumor models. The in vitro nature of the study also precludes the assessment of treatment responses in an in vivo tumor environment. Moreover, the relatively small number of biological replicates in some experiments may affect statistical power to detect smaller treatment effects. Finally, the range of drug concentrations investigated was limited and may not capture the full spectrum of treatment responses. Given the molecular heterogeneity of HCC, validation in additional HCC models with distinct molecular backgrounds, multicellular 3D systems, patient-derived HCC organoids, and in vivo models will be required.

Although 3D models, of course, cannot fully recapitulate the complexity of the in vivo tumor microenvironment, their three-dimensional architecture may better capture aspects of tumor-associated drug responses than conventional 2D cultures such as spatial organization and cellular interactions. These characteristics make 3D cultures a more physiologically relevant system for studying HCC cell behavior and treatment responses than conventional 2D monolayers [108]. Although spheroids may exhibit lower apparent sensitivity to cytotoxic treatments, this feature may better reflect clinically relevant resistance mechanisms rather than represent a limitation of the model.

Consistent with this rationale, our data showed comparable drug effects across 2D and 3D culture systems, supporting the utility of spheroid models for the preclinical evaluation of therapeutic approaches in HCC. Translationally, the integration of 3D culture systems into drug-testing workflows may enhance the identification of treatment strategies that remain effective in a tumor-like microenvironment. Such models may therefore contribute to more predictive preclinical screening, improved prioritization of drug combinations, and a more reliable bridge between in vitro findings and clinical application.

## 5. Conclusions

In summary, the combination of sorafenib or regorafenib with epigenetic or metabolic targeting agents increased cell death and reduced cellular energy levels in HepG2 hepatoma cells compared with the corresponding single-agent treatments. Notably, 3D-cultured HepG2 cells exhibited reduced sensitivity to sorafenib, regorafenib, panobinostat, and NCT-503 compared with 2D cultures, underscoring the impact of culture dimensionality on treatment response. These findings support the use of 3D culture models as a complementary approach to 2D systems for evaluating drug responses, particularly given their ability to capture treatment tolerance not observed to the same extent in 2D cultures. From a translational perspective, 3D models may therefore serve as a more predictive platform for evaluating drug combinations and identifying therapeutic strategies with greater relevance for HCC treatment.

## Figures and Tables

**Table 1:** 2D vs. 3D culture effects on cell death.

Sora	Rego	Pano	NCT	LSMean 2D	LSMean 3D	Δ (2D−3D)	Slice *p*	Interpretation
Control				−0.03	0.03	−0.06	0.992	No difference
5				17.00	10.40	+6.60	0.263	No difference
10				14.33	17.03	−2.70	0.646	No difference
**5**			**40**	**25.07**	**12.43**	**+12.63**	**0.0349**	**↑ Cell death in 2D**
5			80	30.57	22.70	+7.87	0.183	No difference
	5			27.90	16.60	+11.30	0.058	Trend ↑ in 2D
	10			24.97	21.77	+3.20	0.586	No difference
	**5**		**40**	**35.00**	**11.23**	**+23.77**	**0.0002**	**↑ Cell death in 2D**
	**5**		**80**	**46.23**	**28.87**	**+17.37**	**0.0044**	**↑ Cell death in 2D**
		**0.02**		**60.10**	**30.73**	**+29.37**	**<0.0001**	**↑ Cell death in 2D**
		0.5		72.20	71.60	+0.60	0.919	No difference
	**5**	**0.02**		**74.20**	**55.00**	**+19.20**	**0.0018**	**↑ Cell death in 2D**
**5**		**0.02**		**65.23**	**37.47**	**+27.77**	**<0.0001**	**↑ Cell death in 2D**
			40	2.67	2.87	−0.20	0.973	No difference
			80	6.70	8.30	−1.60	0.785	No difference

Sora = sorafenib (µM), Rego = regorafenib (µM), Pano = panobinostat (µM), NCT = NCT-503 (µM). Slice *p*-values are unadjusted and derived from exploratory post hoc slice tests following a significant global culture × treatment interaction (*p* = 0.0001). After Holm correction for 15 comparisons, all differences except sorafenib (5 µM) + NCT-503 (40 µM) remained statistically significant. Bold indicates statistically significant differences between 2D and 3D cultures, with increased cell death in 2D cultures.

## Data Availability

The original contributions presented in this study are included in the article/Supplementary Materials. Further inquiries can be directed at the corresponding author.

## Supplementary Materials

The following supporting information can be downloaded at: https://www.mdpi.com/article/10.3390/biomedicines14102326/s1, Figure S1: Induction of cell death in 2D cultured HepG2 cells upon treatment with kinase inhibitors and epigenetic or metabolic modulators; Figure S2: Induction of cell death in 3D cultured HepG2 cells upon treatment with kinase inhibitors and epigenetic or metabolic modulators; Figure S3: Energy depletion in 2D cultured HepG2 cells upon treatment with kinase inhibitors and epigenetic or metabolic modulators; Figure S4: Energy depletion in 3D cultured HepG2 spheroids upon treatment with kinase inhibitors and epigenetic or metabolic modulators.
