# Supplementary material for: Culture Dimensionality Shapes Drug Sensitivity to Kinase Inhibitor Combinations with Epigenetic or Metabolic Inhibition in a Hepatocellular Carcinoma Model
Source: Biomedicines. 2026 Oct 13;14(10):2326. doi: 10.3390/biomedicines14102326 (PMC13701745; doi:10.3390/biomedicines14102326)
Supplement: Supplementary file 1 [file biomedicines-14-02326-s001.zip › biomedicines-4424738-supplementary.pdf]

Supplementary Materials

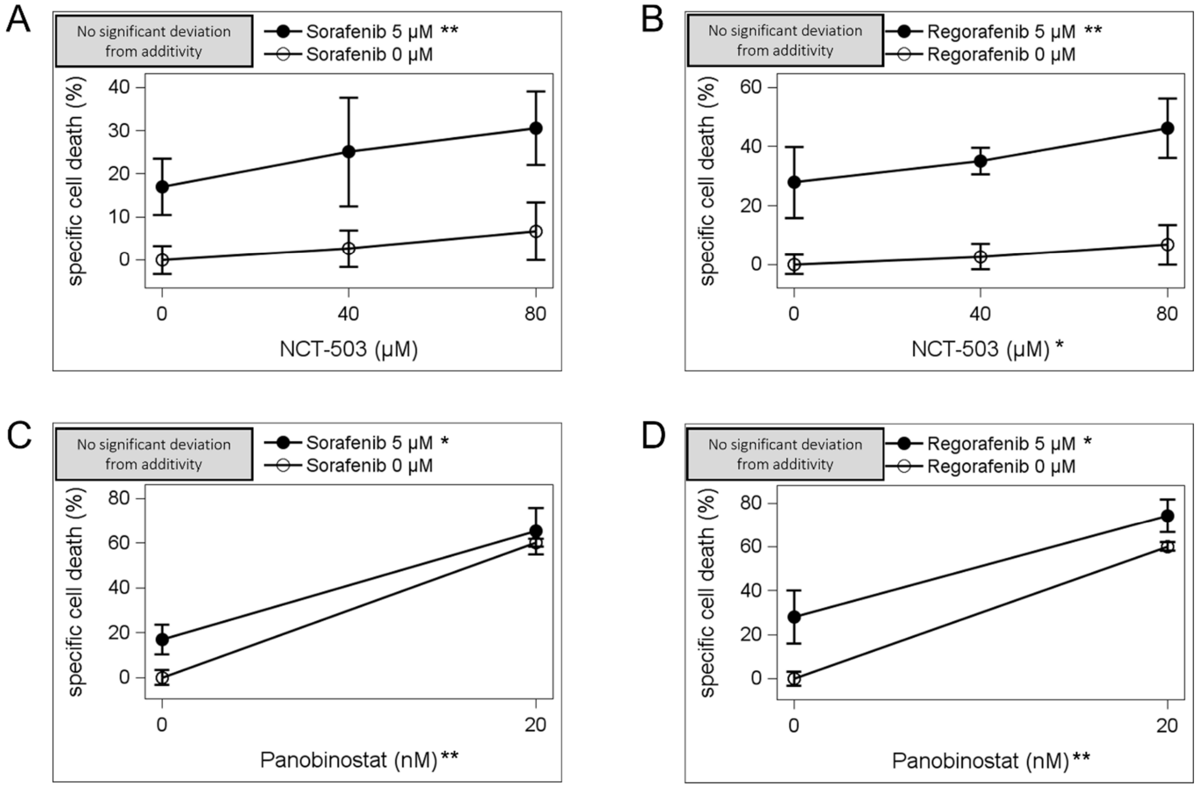

Figure S1. Induction of cell death in 2D cultured HepG2 cells upon treatment with kinase inhibitors and epigenetic or metabolic modulators. HepG2 cells were cultured under 2D conditions and treated with Sorafenib, Regorafenib, NCT-503, and Panobinostat or their combinations for 48 h. Specific cell death was determined by flow cytometry after Annexin V/DAPI staining (n = 3). Solvent-treated cells (corresponding concentration of DMSO) served as control. (A-D) Formal interaction and difference-in-differences (DiD) analyses were conducted to evaluate potential synergistic interactions between kinase inhibitors and epigenetic or metabolic modulators. (A) ** = Type III Test Sorafenib effect: $p < 0.0001$; Sorafenib x NCT-503 interaction showed no significant deviation from additivity: $p = 0.72$. (B) * = Type III Test NCT-503 effect: $p = 0.0415$, ** = Type III Test Regorafenib effect: $p < 0.0001$; Regorafenib x NCT-503 interaction showed no significant deviation from additivity: $p = 0.43$. (C) ** = Type III Test Panobinostat effect: $p < 0.0001$, * = Type III Test Sorafenib effect: $p = 0.0175$; Sorafenib x Panobinostat interaction showed no significant deviation from additivity: $p = 0.15$. (D) * = Type III Test Regorafenib effect: $p = 0.0011$, ** = Type III Test Panobinostat effect: $p < 0.0001$; Regorafenib x Panobinostat interaction showed no significant deviation from additivity: $p = 0.14$.

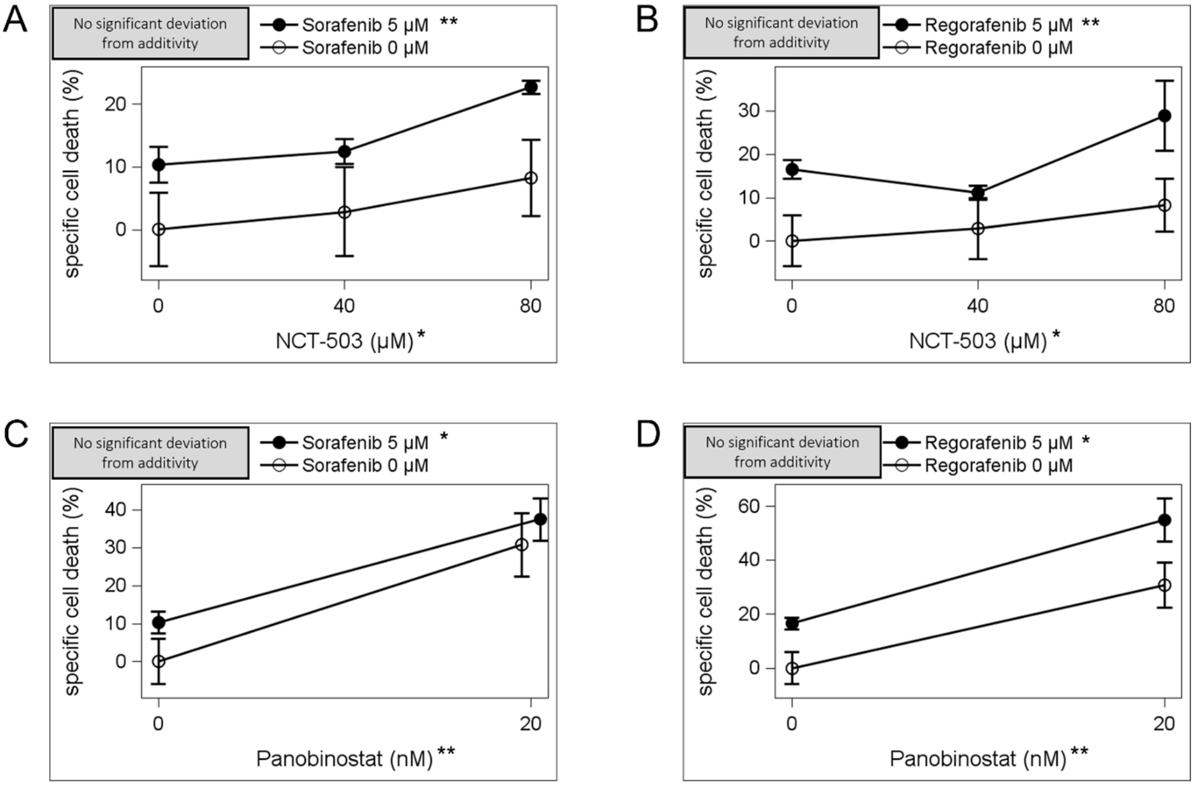

Figure S2. Induction of cell death in 3D cultured HepG2 cells upon treatment with kinase inhibitors and epigenetic or metabolic modulators. HepG2 cells were cultured under 3D conditions as spheroids and treated with Sorafenib, Regorafenib, NCT-503, and Panobinostat or their combinations for 48 h. Specific cell death was determined by flow cytometry after Annexin V/DAPI staining (n = 3). Solvent-treated cells (corresponding concentration of DMSO) served as control. (A-D) Formal interaction and difference-in-differences (DiD) analyses were conducted to evaluate potential synergistic interactions between kinase inhibitors and epigenetic or metabolic modulators. (A) * = Type III Test NCT-503 effect: $p = 0.007$, ** = Type III Test Sorafenib effect: $p = 0.0003$; Sorafenib x NCT-503 interaction showed no significant deviation from additivity: $p = 0.65$. (B) * = Type III Test NCT-503 effect: $p = 0.008$, ** = Type III Test Regorafenib effect: $p = 0.0001$; Regorafenib x NCT-503 interaction showed no significant deviation from additivity: $p = 0.21$. (C) * = Type III Test Sorafenib effect: $p = 0.0395$, ** = Type III Test Panobinostat effect: $p < 0.0001$; Sorafenib x Panobinostat interaction showed no significant deviation from additivity: $p = 0.615$. (D) * = Type III Test Regorafenib effect: $p = 0.0007$, ** = Type III Test Panobinostat effect: $p < 0.0001$; Regorafenib x Panobinostat interaction showed no significant deviation from additivity: $p = 0.34$.

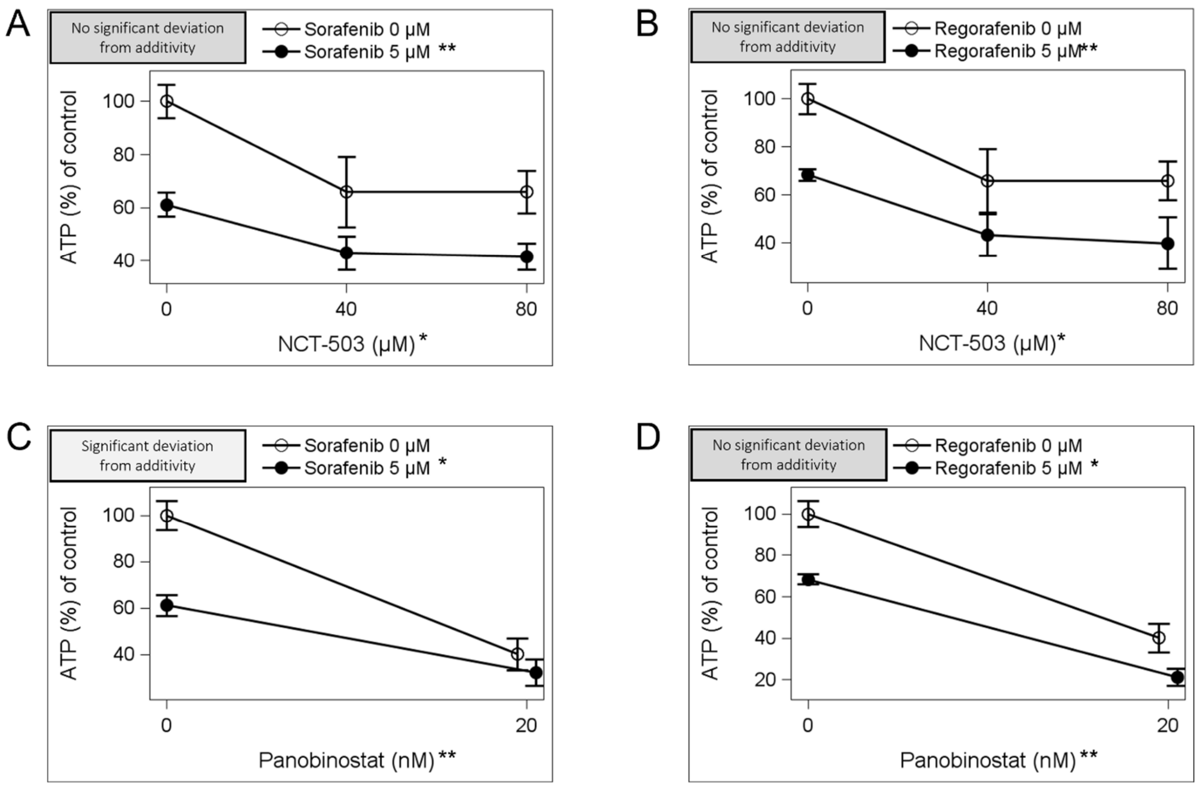

Figure S3. Energy depletion in 2D cultured HepG2 cells upon treatment with kinase inhibitors and epigenetic or metabolic modulators. HepG2 cells were cultured under 2D conditions and treated with Sorafenib, Regorafenib, NCT-503, and Panobinostat or their combinations for 48 h. Cellular energy levels were determined using an ATP assay (n = 4). Solvent-treated cells (corresponding concentration of DMSO) served as control. (A-D) Formal interaction and difference-in-differences (DiD) analyses were conducted to evaluate potential synergistic interactions between kinase inhibitors and epigenetic or metabolic modulators. (A) * = Type III Test NCT-503 effect: $p < 0.0001$, ** = Type III Test Sorafenib effect: $p < 0.0001$; Sorafenib x NCT-503 interaction showed no significant deviation from additivity: $p = 0.6617$. (B) * = Type III Test NCT-503 effect: $p < 0.0001$, ** = Type III Test Regorafenib effect: $p < 0.0001$; Regorafenib x NCT-503 interaction showed no significant deviation from additivity: $p = 0.88$. (C) * = Type III Test Sorafenib effect: $p = 0.0085$, ** = Type III Test Panobinostat effect: $p < 0.0001$; Sorafenib x Panobinostat interaction showed significant deviation from additivity: $p = 0.0409$. This was further supported by a significant DiD contrast, demonstrating that the ATP reduction induced by Panobinostat was significantly greater in the presence of Sorafenib than expected from the sum of individual effects (DiD estimate = +28.44%, 95% CI 1.39–55.50, $p = 0.0409$). (D) * = Type III Test Regorafenib effect: $p = 0.0011$, ** = Type III Test Panobinostat effect: $p < 0.0001$; Regorafenib x Panobinostat interaction showed no significant deviation from additivity: $p = 0.26$.

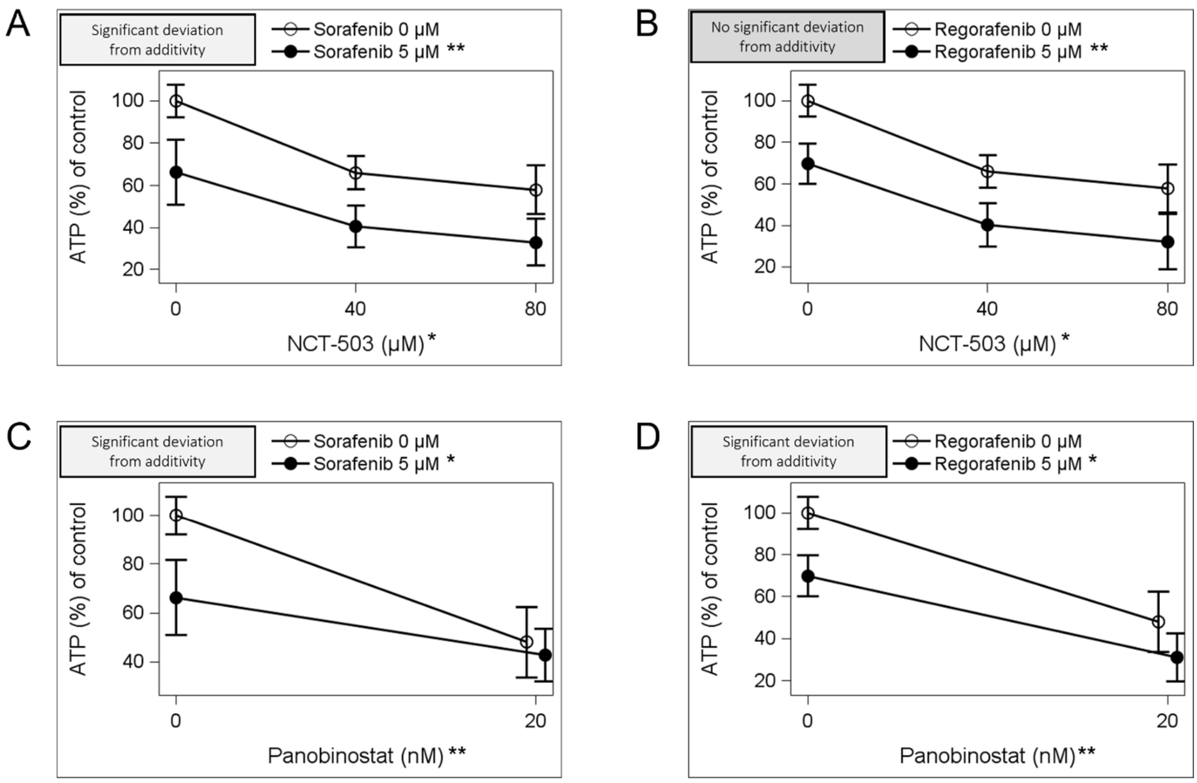

Figure S4. Energy depletion in 3D cultured HepG2 spheroids upon treatment with kinase inhibitors and epigenetic or metabolic modulators. HepG2 cells were cultured under 3D conditions as spheroids and treated with Sorafenib, Regorafenib, NCT-503, and Panobinostat or their combinations for 48 h. Cellular energy levels were determined using an ATP assay ($n = 5$). Solvent-treated cells (corresponding concentration of DMSO) served as control. (A-D) Formal interaction and difference-in-differences (DiD) analyses were conducted to evaluate potential synergistic interactions between kinase inhibitors and epigenetic or metabolic modulators. (A) * = Type III Test NCT-503 effect: $p < 0.0001$, ** = Type III Test Sorafenib effect: $p < 0.0001$; Sorafenib x NCT-503 interaction showed no significant deviation from additivity: $p = 0.0609$. However, directed DiD contrasts demonstrated significant deviations from additivity. Specifically, the ATP-lowering effect of NCT-503 was significantly attenuated in the presence of Sorafenib at both 40 µM (DiD = +15.8, $p = 0.0327$) and 80 µM (DiD = +14.5, $p = 0.0495$). These positive DiD estimates indicate that the combined ATP reduction was less pronounced than expected under a purely additive model. (B) * = Type III Test NCT-503 effect: $p < 0.0001$, ** = Type III Test Regorafenib effect: $p < 0.0001$; Regorafenib x NCT-503 interaction showed no significant deviation from additivity: $p = 0.54$. (C) * = Type III Test Sorafenib effect: $p < 0.0001$, ** = Type III Test Panobinostat effect: $p < 0.0001$; Sorafenib x Panobinostat interaction showed highly significant deviation from additivity: $p < 0.0001$. DiD analysis confirmed this finding, demonstrating a significant positive DiD estimate (+30.9, $p < 0.0001$), consistent with an antagonistic interaction with Sorafenib diminishing additional ATP-reducing effects of Panobinostat. (D) * = Type III Test Regorafenib effect: $p < 0.0001$, ** = Type III Test Panobinostat effect: $p < 0.0001$; Regorafenib x Panobinostat interaction showed significant deviation from additivity: $p = 0.0156$. Panobinostat-associated ATP decrease was significantly larger under regorafenib treatment than under regorafenib-free conditions, yielding a positive and statistically significant DiD (+12.68, $p = 0.0156$).
